# Retinal and choroidal thickness in paediatric patients with hypoalbuminaemia caused by nephrotic syndrome

**DOI:** 10.1186/s12886-019-1050-0

**Published:** 2019-02-06

**Authors:** Wenbo Zhang, Yadi Zhang, Lei Kang, Xiaopeng Gu, Hailong Wu, Liu Yang

**Affiliations:** Department of Ophthalmology, Peking University Frist Hospital, No. 8 Xishiku Street, Xicheng District, Beijing, 100034 China

**Keywords:** Nephrotic syndrome, Hypoalbuminaemia, Retinal thickness, Choroidal thickness

## Abstract

**Background:**

A study was conducted to evaluate the choroidal thickness (CT) and retinal thickness (RT) in paediatric patients with hypoalbuminaemia caused by nephrotic syndrome (NS). We also studied the correlation between the subfoveal choroidal thickness (SFCT) and serum protein concentration.

**Methods:**

This was a cross-sectional study. Fifty-one paediatric patients with hypoalbuminaemia caused by NS and 41 normal subjects were included in the study. Enhanced depth imaging optical coherence tomography (EDI-OCT) was performed to measure the RT and CT. The RT and CT were measured manually at intervals of 0.5 mm along a horizontal line through the macular fovea between 2.5 mm nasal and 2.5 mm temporal to the fovea. Clinical data including measurements of serum proteins were obtained.

**Results:**

The mean RTs at the T2.5, T2, N1.5, N2, and N2.5 locations and the average macular horizontal RT were slightly greater in the NS group than those in the control group. The mean CTs at all locations were significantly greater in the NS group than those in the control group; the difference was most significant at the fovea (373.8 ± 74.9 μm vs. 280.2 ± 57.1; *p* < 0.001). The SFCT in patients with NS was correlated with age (*r* = − 0.307, *p* = 0.003), body height (*r* = − 0.320, *p* = 0.022), body weight (*r* = − 0.343, *p* = 0.014), axial length (AL, *r* = − 0.237, *p* = 0.023), total protein (TP, *r* = − 0.302, *p* = 0.031), albumin (ALB, *r* = − 0.285, *p* = 0.042), prealbumin (PA, *r* = − 0.303, *p* = 0.033) and 24-h urine volume (UV, *r* = − 0.298, *p* = 0.034). Multiple linear regression analysis showed that the TP concentration and body weight had the highest correlation with the SFCT (R^2^ = 0.220, *p* < 0.05).

**Conclusions:**

The macular RT is slightly increased and the macular CT is significantly increased in paediatric patients with hypoalbuminaemia caused by NS, indicating fluid accumulation in the retina and choroid. There is a negative correlation between the SFCT and serum TP concentration. Thus, the serum TP concentration is an important indicator of CT in patients with hypoalbuminaemia.

## Background

Nephrotic syndrome (NS) is a group of clinical syndromes resulting in extensive loss of plasma proteins in the urine due to increased permeability of glomerular basement membrane [1, 2]. A large amount of albumin (ALB) loss in the urine leads to hypoalbuminaemia and decreases plasma colloid osmotic pressure; thus, liquid can enter into the interstitial space from the vascular lumen, causing oedema formation. NS is a important chronic disease of children, and the incidence of NS is reported to be approximately 1–3 per 100,000 children worldwide [1].

In recent years, some case reports have shown that patients with NS can have bilateral serous retinal detachment (SRD) [3–8], macular oedema [3, 8, 9] and retinal pigment epithelium (RPE) detachment [4], accompanied by an increased choroidal thickness (CT) [6]. SRD and choroidal thickening may also occur in patients with hypoproteinaemia caused by other diseases, such as protein losing enteropathy [7, 10]. Therefore, it is presumed that the pathogenesis may be related to the retention of liquid in the retina and choroid due to hypoproteinaemia, which is the same mechanism that leads to systemic oedema [5, 6, 8]. However, there is no research about the effect of hypoproteinaemia on the retinal thickness (RT) and the CT.

Optical coherence tomography (OCT) has rapidly developed in recent years, providing a better opportunity to observe ocular tissues, such as the optic disc and the retina [11]. With the advent of enhanced depth imaging optical coherence tomography (EDI-OCT), measurement of the CT in vivo has become possible [12]. NS is common in children, but poor understanding of the visual impairments in children impedes the early diagnosis of ocular complications. In this study, we examined the CT and RT in paediatric patients with hypoalbuminaemia caused by NS using EDI-OCT to explore the effect of hypoproteinaemia on the RT and CT and discussed the pathogenesis of macular oedema and SRD caused by hypoalbuminaemia.

## Methods

### Subjects

This was a single-centre, cross-sectional, clinical observational study. The study complied with the principles of the Declaration of Helsinki and was approved by the National Unit of Clinical trial Ethics Committee, Peking University First Hospital. Written informed consent was obtained from all the parents/legal guardians of the participants. Paediatric patients who were diagnosed with primary NS at Peking University First Hospital between May 2017 to March 2018 were included in this study according to the following diagnostic criteria: heavy proteinuria (urinary protein excretion ≥50 mg/kg per 24 h, urine protein/creatinine ≥2 mg/mg, or 3+ to 4+ protein in the urine at least 3 times a week) and serum albumin < 25 g/L [2]. Patients who were experiencing the first onset of NS, who relapsed, who were undergoing treatment for NS and who were not cured were all included. Patients had a serum ALB concentration lower than 38 g/L (the normal value in children is between 38 and 54 g/L) [13]. Age- and sex-matched healthy children were evaluated as a control group. The exclusion criteria included (1) refractive errors greater than ±3.0 dioptres; (2) severe cataracts; (3) glaucoma that was not well controlled by drugs; (4) retinal, choroidal, or optic nerve pathology; (5) uveitis, ocular inflammatory diseases, or active or recent infection; (6) a history of intraocular surgery or ocular trauma; (7) secondary NS, severe heart and liver dysfunction, diabetes, autoimmune diseases, hereditary metabolic diseases or haematologic diseases.

### Data collection

A detailed clinical history was obtained, and a full ophthalmologic examination was performed in each subject, including measurements of the best-corrected visual acuity (BCVA) and noncontact intraocular pressure (IOP), slit-lamp examination of the anterior segment, dilated fundus examination by indirect ophthalmoscopy, measurement of the axial length (AL) with an IOL Master 500 (Carl Zeiss Meditec, Jena, Germany) and EDI-OCT (Heidelberg Engineering, Heidelberg, Germany). The body height and weight were recorded. The following clinical data was also collected from patients: total protein (TP), ALB, prealbumin (PA), serum creatinine, uric acid, blood urea nitrogen, triglycerides, total cholesterol, high-density lipoprotein cholesterol, low-density lipoprotein cholesterol, 24-h urine volume (UV), urine protein concentration, 24-h urine total protein, urine creatinine, and 24-h creatinine clearance rate.

**Fig. 1:**
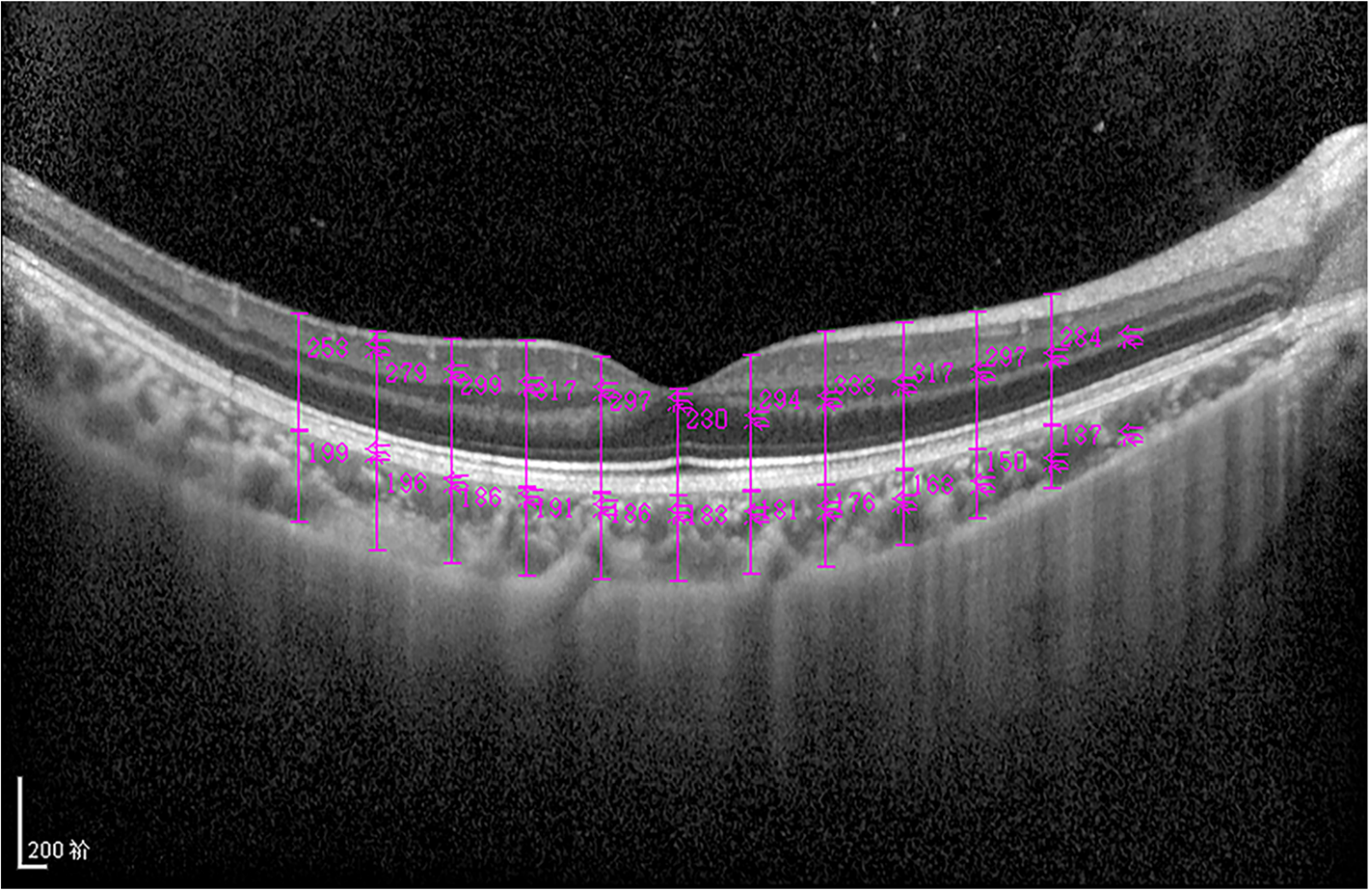
Measurement of RT and CT. RT was measured manually from the inner boundary of the retina to the outer boundary of the RPE, and CT was measured from the outer boundary of the RPE to the inner boundary of the sclera at 0.5-mm intervals along a horizontal line through the macular fovea beginning at the position 2.5 mm to the temporal side of the fovea to the position 2.5 mm to the nasal side of the fovea

EDI-OCT examinations were performed at the same time of day (between 2:00 and 4:00 PM). All subjects were examined by an experienced technician after pupil dilation with 0.5% phenylephrine and 0.5% tropicamide. One high-quality horizontal macular image through the fovea superposed with 100 images was obtained from all subjects. Measurements of RT and CT were performed according to Yan’s method as described previously [14]. In brief, the RT was measured manually from the inner boundary of the retina to the outer boundary of the RPE, and the CT was measured from the outer boundary of the RPE to the inner boundary of the sclera using measurement software provided by Heidelberg. The RT and CT were measured at intervals of 0.5 mm beginning at the position 2.5 mm to the temporal side of the fovea to the position 2.5 mm to the nasal side of the fovea (Fig. 1). N0.5 was used to represent the position 0.5 mm from the nasal side of the fovea, and T0.5 was used to represent the position 0.5 mm from the temporal side of the fovea, and so on. Each location was measured by two operators masked to the group of the patients. The mean macular horizontal RT was obtained by averaging the RTs at all the locations, and the mean macular horizontal CT was obtained by averaging the CTs at all the locations.

### Data analysis

Statistical analysis was performed using SPSS software (version 17.0; IBM Corporation, Armonk, NY, USA). Right eyes of subjects were selected for statistical analysis. The Kolmogorov-Smirnov test was used to assess normality. Continuous variables are expressed as means ± standard deviations and median (min–max) where appropriate. Differences between quantitative parameters were compared by an independent samples *t*-test (normal distribution) or the Mann-Whitney *U*-test (non-normal distribution), and qualitative parameters were evaluated with the chi-square test. Pearson’s correlation tests and stepwise multiple regression analysis were used to determine the correlations between the subfoveal choroidal thickness (SFCT) and clinical data. A *p* value < 0.05 was considered statistically significant.

## Results

Fifty-one patients with NS (mean age of 8.4 ± 3.5 years; 35 boys and 16 girls) and 41 normal subjects (mean age of 8.9 ± 3.2 years; 25 boys and 16 girls) were included in this study. The mean duration of NS was 29.5 ± 33.0 (0.2–120) months, the average serum ALB concentration was 21.0 ± 7.3 (10.2–38) g/L, and the average serum TP concentration was 49.2 ± 8.1 (36.2–66.8) g/L in patients with NS. No significant differences in age, sex, AL, IOP or BCVA were detected between the NS group and the control group (*p* > 0.05, Table 1).

**Table 1:** Clinical characteristics of NS patients and normal subjects

	NS group	Control group	*P*
Age (years)	8.4 ± 3.5	8.9 ± 3.2	0.459^a^
Sex (M/F)	35/16	25/16	0.444^b^
AL (mm)	22.9 ± 1.1	23.0 ± 0.8	0.675^a^
BCVA (Snellen)	1.0 (0.2–1.2)	1.0 (0.8–1.2)	0.161^c^
IOP (mmHg)	18.5 ± 4.0	17.2 ± 2.3	0.060^a^
RT T2.5 (μm)	282.0 ± 17.8	274.0 ± 17.4	**0.035** ^a^
RT T2.0 (μm)	305.4 ± 15.8	294.8 ± 17.8	**0.003** ^a^
RT T1.5 (μm)	325.9 ± 14.7	319.9 ± 14.4	0.054^a^
RT T1.0 (μm)	330.5 ± 16.5	327.0 ± 14.9	0.300^a^
RT T0.5 (μm)	286.5 ± 20.5	287.7 ± 20.1	0.775^a^
RT SF (μm)	217.3 ± 16.6	213.0 ± 19.5	0.254^a^
RT N0.5 (μm)	288.1 ± 22.4	289.4 ± 22.5	0.780^a^
RT N1.0 (μm)	345.8 ± 15.3	341.0 ± 15.4	0.144^a^
RT N1.5 (μm)	354.5 ± 14.2	345.8 ± 15.8	**0.006** ^a^
RT N2.0 (μm)	339.8 ± 17.9	330.6 ± 15.1	**0.010** ^a^
RT N2.5 (μm)	319.3 ± 17.5	310.5 ± 17.4	**0.018** ^a^
RT AVG (μm)	308.6 ± 11.9	303.0 ± 12.2	**0.029** ^a^
CT T2.5 (μm)	326.3 ± 75.3	266.1 ± 42.2	**< 0.001** ^a^
CT T2.0 (μm)	342.8 ± 76.8	271.5 ± 40.9	**< 0.001** ^a^
CT T1.5 (μm)	355.5 ± 76.2	276.20 ± 43.1	**< 0.001** ^a^
CT T1.0 (μm)	366.8 ± 73.4	277.5 ± 47.0	**< 0.001** ^a^
CT T0.5 (μm)	372.6 ± 73.6	283.2 ± 52.5	**< 0.001** ^a^
CT SF (μm)	373.8 ± 74.9	280.2 ± 57.1	**< 0.001** ^a^
CT N0.5 (μm)	356.6 ± 75.0	262.9 ± 58.6	**< 0.001** ^a^
CT N1.0 (μm)	333.9 ± 75.1	249.0 ± 58.4	**< 0.001** ^a^
CT N1.5 (μm)	309.9 ± 73.1	227.5 ± 58.7	**< 0.001** ^a^
CT N2.0 (μm)	277.7 ± 67.9	205.1 ± 60.8	**< 0.001** ^a^
CT N2.5 (μm)	246.4 ± 61.6	176.8 ± 57.8	**< 0.001** ^a^
CT AVG (μm)	332.9 ± 66.3	252.4 ± 45.4	**< 0.001** ^a^

Data are expressed in mean ± SD, except for sex and BCVA. BCVA are expressed in median (min–max). Significant differences are highlighted in bold print

*M* male, *F* female, *AL* axial length, *BCVA* best-corrected visual acuity, *IOP* intraocular pressure, *RT* retinal thickness, *CT* choroidal thickness, *N0.5* nasal 500 μm from the fovea, *N1.0* nasal 1000 μm from the fovea, *N1.5* nasal 1500 μm from the fovea, *N2.0* nasal 2000 μm from the fovea, *N2.5* nasal 2500 μm from the fovea, *SF* subfoveal, *T0.5* temporal 500 μm from the fovea, *T1.0* temporal 1000 μm from the fovea, *T1.5* temporal 1500 μm from the fovea, *T2.0* temporal 2000 μm from the fovea, *T2.5* temporal 2500 μm from the fovea, *RT AVG* average macular horizontal RT, *CT AVG* average macular horizontal CT

^a^independent samples *t*-test

^b^chi-square test

^c^Mann-Whitney *U*-test

**Fig. 2:**
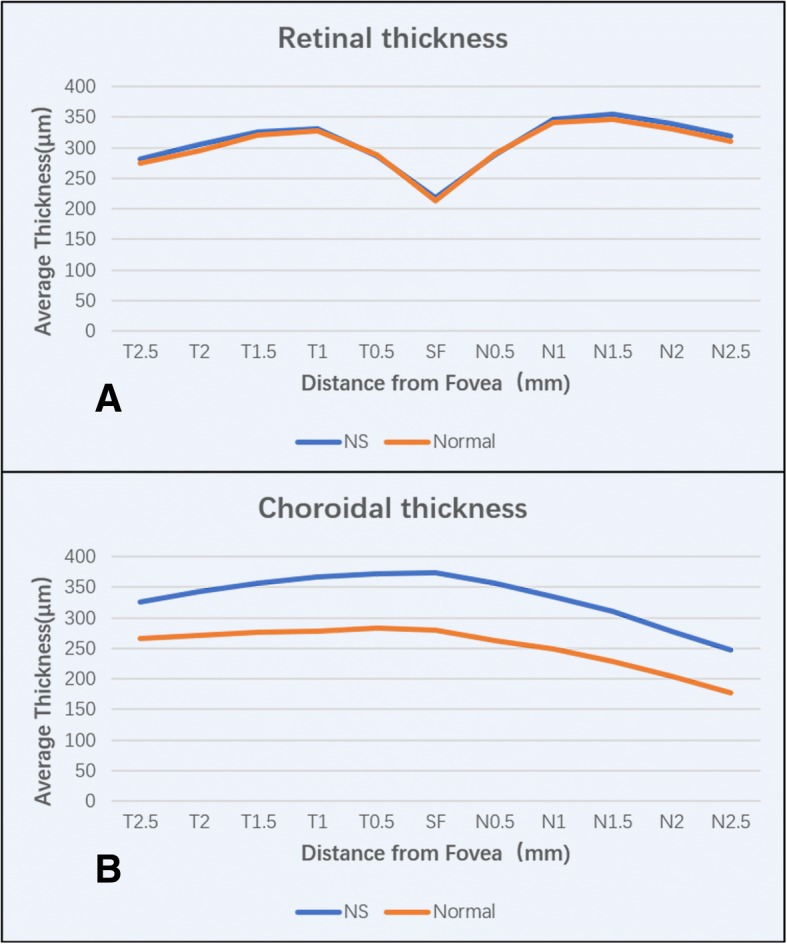
RT (**a**) and CT (**b**) of patients with NS compared with those of normal children

**Fig. 3:**
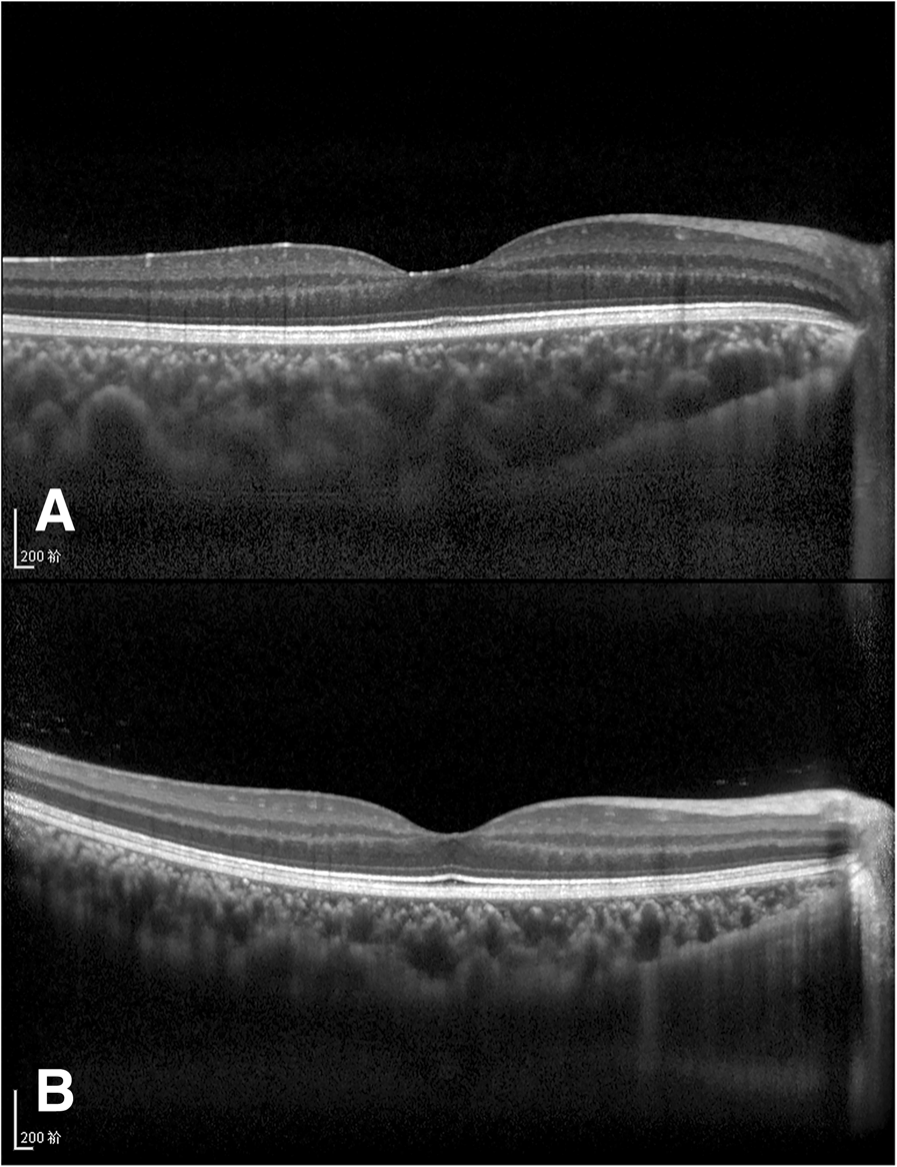
EDI-OCT images from a patient with NS (**a**) and a normal child (**b**). The CT of the NS patient was much greater than that of the normal child

There were no cases of macular oedema or SRD in the subjects. The mean subfoveal retinal thickness of patients in the NS group was 217.3 ± 16.6 μm; the RT was greatest at the N1.5 location and thinnest in the subfoveal area. The mean subfoveal retinal thickness of patients in the control group was 213.0 ± 19.5 μm; the RT was greatest at the N1.5 location and thinnest in the subfoveal area. The mean RTs at the T2.5, T2, N1.5, N2, and N2.5 locations were slightly greater in the NS group than those in the control group (*p* < 0.05). The differences ranged from 8.0 to 10.6 μm. The average macular horizontal RT was slightly greater in the NS group than that in the control group (308.6 ± 11.9 μm vs. 303.0 ± 12.2 μm, *p* < 0.05). No statistically significant differences were detected at other locations (Table 1, Fig. 2a). The mean SFCT of patients in the NS group was 373.8 ± 74.9 μm; the CT was greatest in the subfoveal area and thinnest at the N2.5 location. The mean SFCT of patients in the control group was 280.2 ± 57.1 μm; the CT was greatest at the T0.5 location and thinnest at the N2.5 location. The mean CTs at all locations in the NS group were much greater than those in the control group (*p* < 0.001, Table 1, Fig. 2b). A comparison of EDI-OCT images between a paediatric patient with hypoalbuminaemia caused by NS and a normal child is shown in Fig. 3. The CT of the patient with NS was much greater than that of the normal subject.

A simple linear regression analysis showed that the SFCT was significantly correlated with age, body height, body weight, AL, TP, ALB, PA and 24-h UV (all *p* < 0.05, Table 2). No correlation was found between the SFCT and the duration of the disease, serum creatinine, uric acid, blood urea nitrogen, triglycerides, total cholesterol, high-density lipoprotein cholesterol, low-density lipoprotein cholesterol, urine protein concentration, urine total protein, urine creatinine or 24-h creatinine clearance rate. A stepwise multiple linear regression analysis of the SFCT (Y) by age (X1), body height (X2), body weight (X3), AL (X4), TP (X5), ALB (X6), PA (X7) and UV (X8) was performed. The outcomes showed that the SFCT had the highest correlation with body weight and TP, Y = 597.157–1.873 X3–3.307 X5, determination coefficient (R^2^) = 0.220, (*p* < 0.05).

**Table 2:** Simple regression analysis for the correlations between the SFCT and age, body height, body weight, AL, TP, ALB, PA and 24-h UV in patients with NS

	*r*	*P*
Age (years)	−0.307	**0.003** ^a^
Body height (cm)	−0.320	**0.022** ^a^
Body weight (kg)	−0.343	**0.014** ^a^
AL (mm)	− 0.237	**0.023** ^a^
TP (g/L)	− 0.302	**0.031** ^a^
ALB (g/L)	− 0.285	**0.042** ^a^
PA (mg/L)	− 0.303	**0.033** ^a^
UV (ml)	− 0.298	**0.034** ^a^

Significant differences are highlighted in bold print

*AL* axial length, *TP* total protein, *ALB* albumin, *PA* prealbumin, *UV* urine volume

^a^Pearson’s correlation tests

## Discussion

Renal diseases accompanied by ocular diseases are not rare. They often occur with autoimmune diseases [15], infectious diseases [16], hereditary diseases [17], or metabolic diseases (such as diabetic retinopathy). The most common ocular complications caused by NS are cataracts and glaucoma induced by glucocorticoids [18]. Choroidoretinopathy caused by NS is rare. Among the reported cases, some patients had SRD in the macular region [3–8, 10], while other patients had concurrent macular oedema [3, 8] or RPE detachment and tears [4], or the condition manifested as macular oedema alone [9]. Some patients showed concurrent increases in CT on OCT [6]. Patients suffering from these diseases also typically had hypoalbuminaemia, with blood ALB concentrations of 10–23 g/L, which usually resolved after several weeks of treatment with glucocorticoids, immunosuppressants and diuretics [3–7, 9, 10]. Similarly, SRD and choroidal thickening also occurred in patients with hypoproteinaemia caused by other diseases, such as protein losing enteropathy [7, 10]. Therefore, we presumed that the pathogenesis may be related to the retention of liquid in the retina and choroid due to hypoproteinaemia.

In this study, we used OCT to measure changes in the RT and CT in paediatric patients with hypoalbuminaemia caused by primary NS. In paediatric patients with NS, the CT was greater than that in normal children, and the difference was statistically significant. Nagasawa et al. studied normal paediatric individuals (mean age of 7.9 ± 3.1 years) using OCT and found that the central CT within a 1.0-mm circle was 260.4 ± 57.2 μm [19]. Ruiz-Moreno et al. found that in paediatric individuals (mean age of 10 ± 3 years), the mean SFCT was 312.9 ± 65.3 μm and the average macular horizontal CT was 285.2 ± 56.7 μm [20]. In our study, the mean SFCT was 373.8 ± 74.9 μm, and the average macular horizontal CT was 332.9 ± 66.3 μm in paediatric patients with NS, which were significantly higher than previously reported results. Choroidal thickening caused by NS is associated with hypoproteinaemia. Animal experiments have confirmed that ALB is present in the retinal and choroidal vessels [21]. There are fenestrations (approximately 15 nm in size) in the choroidal vessels, and macromolecules with a molecular weight of 9 × 10^5^ D can pass through them. Therefore, the choroidal interstitial fluid is rich in protein, and the colloid osmotic pressure of the choroidal tissue is high. However, there are no fenestrations in the retinal vessels. Additionally, the vascular endothelium is tightly connected, and the retina has very little interstitial space, thus, the colloid osmotic pressure of the retinal tissue is low [22]. During ALB loss in NS, the colloid osmotic pressure in the choroidal vessels decreases, resulting in the movement of fluid towards the interstitial space of the choroid due to the high colloid osmotic pressure, which leads to fluid accumulation in the interstitial space of the choroid. This is the main cause of choroidal oedema and thickening. Izzedine et al. [6] reported a case of SRD caused by NS (with a TP concentration of 42 g/L and an ALB concentration of 17 g/L) and found increased CT on OCT. Similarly, Venkatramani et al. [10] reported a case of SRD caused by protein losing enteropathy (with an ALB concentration of 14 g/L), and an increased CT was also found on B-mode ultrasound because of the low ALB concentration.

In this study, the mean RTs at the T2.5, T2, N1.5, N2 and N2.5 locations were slightly greater in patients with NS than in normal subjects, and the average macular horizontal RT was also slightly greater in patients with NS than in normal subjects (308.6 ± 11.9 μm vs. 303.0 ± 12.2 μm, *p* < 0.05), indicating fluid accumulation in the retina. This might explain the mechanism of SRD and macular oedema in some patients with hypoalbuminaemia. The inner retinal barrier and outer retinal barrier ensure dehydration of the retina. In addition, the difference in colloid osmotic pressures between the choroid and the retina generates suction that causes fluid to flow from the retina to the choroid and finally to be discharged into the choroidal circulation by the action of the RPE pump [22]. This colloid osmotic pressure difference attaches the retinal neuroepithelial layer to the RPE layer [22, 23]. During ALB loss in NS, increased choroidal interstitial fluid results in a decrease in colloid osmotic pressure, decreasing the pressure difference between the choroid and retina and reducing the flow of fluid from the retina to the choroid, leading to fluid accumulation in the retina and subretinal region [5–7, 10]. This is the main cause of retinal thickening. However, in our study, SRD and macular oedema were not found in any patients. Further, the increased RT at these locations ranged only from 8.0 to 10.6 μm, which was not an obvious thickening and had no effect on BCVA. In the cases of SRD and macular oedema caused by NS reported previously, all the patients were adults; the youngest was a 24-years-old woman [3]. We think that the RPE constantly pumps liquid from the retina into the choroid [22], which can cause retinal oedema only during RPE dysfunction. However, the function of the RPE in children is more precise than that in adults, which makes it difficult for fluid to accumulate in the retina.

Ruiz-Moreno et al. found that in adults, the choroid was the thickest at the fovea, but in paediatric patients, it was the thickest at the temporal side (324 μm), followed by the subfoveal area (312 μm), and it was the thinnest at the nasal side (195 μm) [20]; these findings are in accordance with our results in normal subjects. However, in paediatric patients with NS, the choroid was the thickest at the subfoveal area (373.8 ± 74.9 μm), followed by the temporal side (372.6 ± 73.6 μm) and the nasal side (246.4 ± 61.6 μm). Yan et al. suggested that the choroidal capillary layer at the posterior pole of the macular region is the thickest, and the blood vessel density is the greatest in this region. Therefore, the colloid osmotic pressure in this region is higher than that in the surrounding regions, and the tendency to draw fluid into the choroid is strongest at this location [8, 22]. Thus, hypoproteinaemia has the most direct and serious effect on SFCT. In our study, simple linear regression analysis showed that the SFCT was significantly correlated with age, body height, body weight, AL, TP, ALB, PA and 24-h UV. Nagasawa et al. reported that the central CT within a 1.0 mm circle in normal paediatric individuals was significantly correlated with age, body height, body weight, and AL [19], and these findings are in accordance agree with our results. TP, which includes ALB, globulin and PA, is the main constituent of colloid osmotic pressure in blood vessels and tissues. SFCT was significantly correlated with TP, ALB and PA concentrations, suggesting that the lower the protein concentration in the blood vessels is, the thicker the choroid is. Multiple linear regression analysis showed that the correlation between the TP concentration and SFCT was the highest; when the TP concentration decreased by 1 g/L, the CT increased by 3.307 μm, and when the weight decreased by 1 kg, the CT increased by 1.873 μm (Y = 597.157–1.873 X3–3.307 X5). Thus, in children with NS, the TP concentration and weight are important indicators for measuring CT. We also found that the SFCT was negatively correlated with the 24-h UV. We speculate that the decrease in 24-h UV may aggravate the retention of water and increase the CT.

Patients with NS are prone to concurrent thromboembolism. Masoodi et al. [24] reported a case of NS complicated by renal vein thrombosis and left central retinal vein occlusion. Viola et al. [25] reported a case of NS with Purtscher-like retinopathy after diffuse intravascular coagulation. Shinoda reported a case of NS combined with progressive outer retinal necrosis syndrome due to long-term use of glucocorticoids [26]. However, none of these complications were found in our study.

Our study has some limitations. First, the development of the eyeball and CT vary in children of different ages. We did not stratify the subjects according to age, which may have had some effects on the results. Second, the RT and CT could be influenced by the dioptre and AL. In our study, patients with a refractive error greater than ±3.0 dioptres were excluded. Moreover, no significant differences in AL were found between the NS patients and controls. However, it is not enough. Third, the sample size of the current study was small.

## Conclusions

In conclusion, the macular RT is slightly increased while the macular CT is significantly increased in paediatric patients with hypoalbuminaemia caused by NS, indicating fluid accumulation in the retina and choroid. This might explain the mechanism of SRD and macular oedema in some patients with hypoalbuminaemia. However, there is no effect on the visual acuity of these patients. There is a negative correlation between the SFCT and serum TP concentration. Thus, the serum TP concentration is an important indicator for CT in patients with hypoalbuminaemia.

## Abbreviations

AL: Axial length
ALB: Albumin
BCVA: Best-corrected visual acuity
CT: Choroidal thickness
EDI-OCT: Enhanced depth imaging optical coherence tomography
IOP: Intraocular pressure
NS: Nephrotic syndrome
PA: Prealbumin
RPE: Retinal pigment epithelium
RT: Retinal thickness
SFCT: Subfoveal choroidal thickness
SRD: Serous retinal detachment
TP: Total protein
UV: Urine volume
